# A leakage-aware genomic prediction pipeline for meropenem resistance in *Klebsiella pneumoniae* using transformer-based resistome representation learning

**DOI:** 10.1093/bioadv/vbag193

**Published:** 2026-07-29

**Authors:** İlkay Sibel Kervancı

**Affiliations:** Department of Computer Engineering, Engineering Faculty, Gaziantep University, Gaziantep 27310, Turkey; Department of Bioinformatics and Computational Biology, Graduate School of Health Sciences, Gaziantep University, Gaziantep 27310, Turkey

## Abstract

**Motivation:**

Antimicrobial resistance (AMR) in *Klebsiella pneumoniae*, particularly to carbapenems such as meropenem, is a major global health problem. Machine learning is increasingly used to predict resistance from genomic markers; however, many models fail to capture high-level gene–gene interactions and may exhibit inflated performance due to lineage-biased prediction. Existing genomic prediction models largely rely on flat feature representations that fail to capture epistatic gene interactions, and commonly suffer from inflated performance estimates due to phylogenetic data leakage. To address these limitations simultaneously, a leakage-aware hybrid TabTransformer-CatBoost pipeline was developed, combining self-attention-based resistome representation learning with gradient boosting classification under clade-aware data partitioning. A self-attention encoder converts sparse gene presence–absence profiles into contextualized latent embeddings, which are subsequently classified using gradient boosting to capture lineage-aware AMR patterns.

**Results:**

The proposed architecture outperformed classical baselines including Logistic Regression, Random Forest, XGBoost, and optimized CatBoost models. Internal accuracy reached 92.59% for the Chained Hybrid configuration (area under the receiver operating characteristic curve, AUROC = 0.8670, F1 = 0.8537). Performance gains primarily originated from the embedding stage, as confirmed by ablation analysis. External validation across independent multinational cohorts (*n* = 305) demonstrated generalizability (AUROC = 0.8105; F1 = 0.7552). Permutation testing produced near-zero Matthews Correlation Coefficient (MCC) = 0.0091, indicating predictions reflect genuine biological signal rather than noise. These results establish attention-based genomic embedding with gradient boosting as a scalable, interpretable, and leakage-aware framework for clinical AMR prediction.

**Availability and implementation:**

The source code for the TabTransformer–CatBoost framework, including preprocessing pipelines and pre-trained embeddings, is available at https://github.com/SibelKervanci/kp-meropenem-tabtransformer.

## 1 Introduction

The dramatic pace of spread of antimicrobial resistance (AMR) globally remains a significant obstacle for modern medicine. Many bacterial pathogens have evolved processes that allow them to subsist under antibiotic concentrations, which were earlier effective, resulting in increased morbidity and mortality (Llor and Bjerrum 2014). While AMR is a disease-causing agent of morbidity and mortality across a range of bacteria, the clinical burden of AMR is worse in Gram-negative microorganisms. Of these, for example, *Klebsiella pneumoniae* is an Enterococcus, Staphylococcus, Klebsiella, Acinetobacter, Pseudomonas, Enterobacter (ESKAPE) organism (Li *et al.* 2023) and of these, the ESKAPE group is one of the most challenging with high burden factors; however, it persists to affect a variety of healthcare-associated infections including pneumonia, sepsis, and bloodstream infections. The rising rates of carbapenem-resistant *K.pneumoniae* (CRKP) are of particular concern. With multidrug-resistant infections, carbapenems like meropenem have historically been the last-line agents given to try different types of drugs to treat them. However, plasmid-mediated dissemination of resistance determinants has further eroded their clinical utility (Wyres and Holt 2018). Simultaneously, the development pipeline for new antibiotics has been reduced by both regulatory constraints and underfunding and the reduction of commercial incentives associated with short-course antimicrobial agents in short-course therapies has slowed the development as well as regulations for development of newer antibiotics (Fanelli *et al.* 2020). This means that quick and accurate diagnostic methods are critical. Conventional phenotypic susceptibility testing is the clinical standard (Laxminarayan *et al.* 2013), but usually takes 48–72 h to generate results, often resulting in empirical broad-spectrum treatment followed by selection of resistant strains as appropriate. Whole-genome sequencing (WGS) gives a more rapid and holistic view of pathogen genomes, but translating high-dimensional genomic data into clinically actionable resistance predictions still is computationally complex. Recent investigations emphasize that machine learning methods can solve this issue by making resistance prediction possible from genomics information by employing different encoding methodologies (Ren *et al.* 2022). Transformer-based architectures with structured latent spaces have shown particular promise across diverse bioinformatics tasks, including multi-target molecular generation under data-limited conditions (Norouzi *et al.* 2026). A number of computational tools have shown that combined whole-genome sequencing and machine learning can facilitate high-performance scalable AMR prediction pipelines (Weis *et al.* 2020). Traditional machine learning approaches such as Support Vector Machine (SVM) and Random Forest (RF) have demonstrated strong performance for predicting AMR from genomic data. Recent studies further indicate that hybrid tree-based and deep learning architectures can capture complex feature interactions in high-dimensional genomic datasets, enabling improved detection of resistance determinants (Tan *et al.* 2021, Borisov *et al.* 2024, Orcales *et al.* 2024, Pei *et al.* 2024, Dehghan *et al.* 2026). To this end, a hybrid framework that predicts these determinants but also accounts for their contextual dependencies is applied in building off of these insights. Transformer-based approaches to antimicrobial-resistance prediction were also tested, including the sequence-level models trained on raw nucleotide data (Ji *et al.* 2021), despite the fact that many of these approaches still used clinical metadata instead of direct genomic coding (Tharmakulasingam *et al.* 2023). Although tools such as DeepARG have improved antimicrobial resistance gene recognition based on metagenomic data for resistance gene identification by improving metagenomic data, bag-of-genes approaches cannot capture epistatic interactions between distant genomic markers (Arango-Argoty *et al.* 2018). Genomic data are also vulnerable to information leakage and lineage-based bias, especially when closely related isolates are present both in train and test sets. Despite these advances, existing genomic AMR prediction pipelines rarely address lineage-driven information leakage and higher-order gene–gene interactions simultaneously across heterogeneous cohorts. To address these limitations, we developed a hybrid TabTransformer–CatBoost architecture that models contextual relationships among resistance genes. The TabTransformer encoder transforms sparse gene presence–absence profiles into contextualized latent embeddings using self-attention (Huang *et al.* 2020), which are subsequently classified with a gradient-boosted framework inspired by prior hybrid tabular models (Khalid *et al.* 2025). We then classify learned representations with CatBoost, a gradient-boosted tree model that has shown strong performance in structured and tabular data. Tree-based models in these domains often far exceed pure deep learning architectures, especially when working with tabular genomic datasets that consist of sparse and high-dimensional data (Hancock and Khoshgoftaar 2020, Grinsztajn *et al.* 2022). The model proposes to model non-linear gene-gene interactions and contextual dependencies in features projecting them into a latent space prior to classification that might not be possible with classical classification models (Choi and Lee 2023). Multi-study dataset was used for model development with strict duplicate-free deletion to eliminate the same genomic profiles (i.e. a set of genetically identical isolates with conflicting phenotypes) on the data. To resist phylogenetic leakage, isolates were divided by the clade-aware ensemble on the basis of the Hamming distance in gene presence–absence profiles. This approach ensures that closely related genomes do not appear simultaneously in both training and test sets, making model generalization a more realistic metric. We propose a hybrid method based on a sequential pipeline which unites attention-based feature extraction and gradient-boosted classification. This architecture also corresponds with the practical finding that robust genomics-based embeddings enhance subsequent classifications performance while remaining interpretable and stable during training. In this study, we mainly focus on assessing the ability to use embedding-based genomic representations to enhance resistance-prediction robustness over heterogeneous cohorts. We find particularly whether attention-derived latent features improve model generalization over time beyond the single dataset in cases of clade-aware splitting and independent external validation. To evaluate reliability even further, permutation testing was conducted as an additional check in order to find if predictive performance in this type of analysis is an authentic genomic signal instead of random structure. To our knowledge few studies have been done that investigated the combination of attention-based genomic embedding models, paired with gradient-boosted classifier approaches for predicting carbapenem resistance in *K.pneumoniae* specifically in the form of heavy clade-aware evaluation and external multi-cohort validation.

The present study addresses three specific gaps in the existing literature:

- The absence of transformer-based resistome representation learning for carbapenem resistance prediction.
- The lack of leakage-aware evaluation protocols that account for phylogenetic structure in genomic AMR datasets.
- The limited external validation of genomic prediction models across geographically diverse multinational cohorts.

To the best of our knowledge, this is the first study to integrate transformer-based resistome representation learning with a leakage-aware genomic partitioning strategy for meropenem resistance prediction in *K.pneumoniae*, combining self-attention-based epistatic encoding, clade-aware data splitting, and multi-objective gradient boosting optimization within a single reproducible pipeline.

The primary contributions of this article are summarized as follows:

1. Unlike sequence-level transformer models that operate on raw nucleotide sequences, our TabTransformer-based pipeline performs resistome representation learning by modeling the presence–absence patterns of 195 curated AMR determinants, enabling the discovery of contextual gene interactions underlying AMR.
2. Unlike conventional AMR prediction studies that rely on random train–test splitting, we introduce a leakage-aware genomic prediction pipeline that integrates transformer-based feature embedding with gradient boosting and multi-objective optimization. The framework incorporates conflict filtering, genomic profile deduplication, and clade-aware partitioning to prevent lineage-driven information leakage and ensure that model evaluation reflects genuine biological signals rather than memorization of clonal signatures.
3. By prioritizing the Matthews Correlation Coefficient (MCC) as the primary evaluation metric and employing multi-objective optimization, the proposed framework improves recall and F1-score to minimize clinically critical false negatives in AMR detection.

## 2 Methods

### 2.1 Dataset description and preprocessing

**Figure 1:**
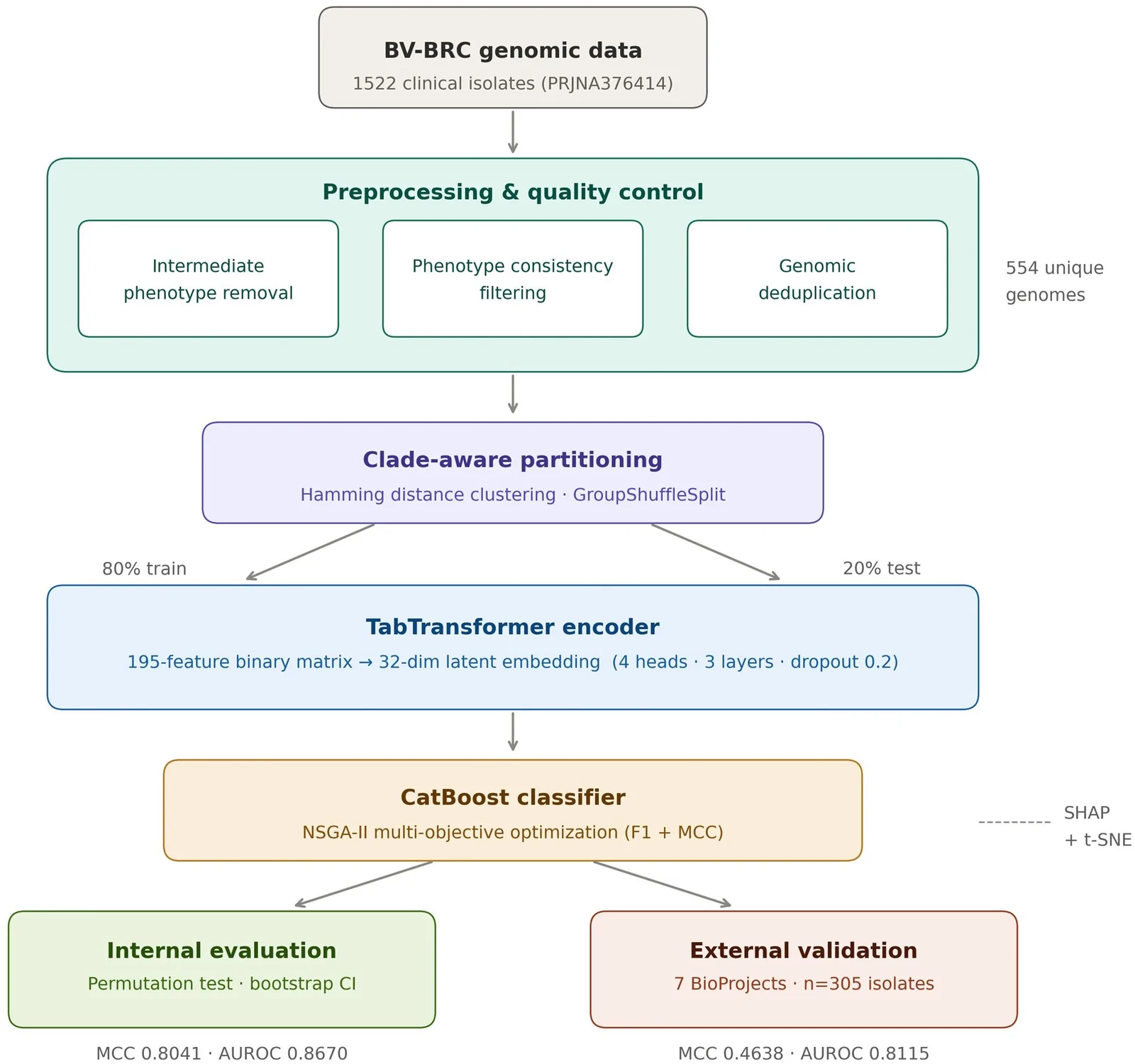
Leakage-aware resistance prediction pipeline. Genomic data were preprocessed through phenotype filtering and deduplication (554 unique genomes), partitioned via clade-aware splitting, encoded into 32-dimensional latent embeddings using a TabTransformer self-attention encoder, and classified with a NSGA-II-optimized CatBoost model. Performance was assessed internally and validated across seven independent BioProjects (*n = *305).

An overview of the proposed hybrid resistance prediction pipeline, including preprocessing, embedding generation, model training, and validation strategy, is shown in Fig. 1.

The genomic dataset used in this article is collected from Bacterial and Viral Bioinformatics Resource Center (BV-BRC) (Olson *et al.* 2023). The major discovery isolates are from the Houston Methodist Hospital clinical data and are listed under the National Center for Biotechnology Information (NCBI) BioProject PRJNA376414 (Long *et al.* 2017b). Our study investigates *K.pneumoniae* (Taxon ID: 573), with an initial dataset containing 1522 clinical isolates. Meropenem resistance was defined with CLSI standardized phenotypic susceptibility data. After excluding isolates of “intermediate” phenotypes (*n* = 45), a discovery cohort of 1448 genomes was established. A rigorous quality control (QC) filter was then used to eliminate another 180 isolates creating a final number of 1268 genomes ready for further analysis. Many of these were eliminated due to complete loss of genomic markers in their profiles, demonstrating either incomplete sequence data or technical issues within feature extraction. In addition, in conformity with prior genomic audits of the Houston cohort, many isolates were excluded after performing taxonomic re-verification, identifying them as non-target species, e.g. *Klebsiella variicola* or *Klebsiella quasipneumoniae*. Purged samples were obtained with highly fragmented assemblies or mismatched SRA records to guarantee that only high-fidelity *K.pneumoniae* (Taxon ID: 573) genomes were employed, in accordance to existing pipeline validation protocols for carbapenem-resistant *K.pneumoniae* (Long *et al.* 2017a, Vieira *et al.* 2023). In order to maintain model validity against noise from information leakage at the time of artificial optimization and to reduce the threat of artificial performance inflation, a stringent multi-stage preprocessing protocol has been developed. The feature space was carefully limited to 195 genomic markers, mainly reflecting classical AMR determinants, including blaKPC variants, porin mutations, and efflux pump entities. For each isolate within this framework, a genomic signature was created by concatenating the binary states of presence-absence of all 195 features into a single vector. After extracting features, we conducted Phenotype Consistency Filtering to remove the label noise: any genomic signature associated with discordant meropenem resistance labels in which identical genotypes displayed as resistant and susceptible among these isolates was methodically removed. Finally, strict genomic deduplication was performed to keep only one instance of each unique, consistent signature representative. The refinement was conducted to create a high-fidelity discovery dataset of 554 distinct genomes, that included 413 resistant and 141 susceptible isolates. Thus prioritizing these genomic signatures over raw isolate counts forced the architecture to learn genuine epistatic interactions, rather than mere pattern memorization of repetitive sequences and significant sequences in clonal expansion, thus setting the stage for a conservative and biologically legitimate system of evaluation. A comprehensive description of cohort constitution before and after deduplication is given in Table 1.

**Table 1:** Composition of discovery and external validation cohorts before and after genomic deduplication.

Cohort	Samples	Resistant	Susceptible
Training	1268	335	933
Training (unique)	554	141	413
External	305	184	121
External (unique)	199	135	64

### 2.2 Independent external validation cohort

A separate independent external validation set was created to assess global generalizability of the framework based on seven separate NCBI BioProjects not included during the initial discovery stage. This cohort initially consisted of 305 isolates that were exposed to the same stringent deduplication and phenotypic-consistency filtering protocols used in the discovery dataset, yielding 199 unique genomic profiles. We included isolates from BioProject PRJEB6574 (*n = *108) (Martin *et al.* 2016) and PRJNA288601 (*n = *113), PRJNA278886 (*n = *31), PRJNA307517 (*n = *22), PRJNA296771 (*n = *15), PRJEB22890 (*n = *11), and PRJNA312972 (*n = *5). All isolates were curated according to the standard AMR phenotype prediction framework developed by the PATRIC/BV-BRC infrastructure (Davis *et al.* 2016). This multi-center validation design evaluates model generalizability across diverse geographic cohorts. No post hoc calibration of probabilities (Platt scaling or isotonic regression) was applied; instead, a fixed threshold derived from theoretical F1 optimization was used to maintain consistent operating characteristics across cohorts.

### 2.3 Model development and evaluation strategy

For model development, the deduplicated discovery dataset (*n = *554) was partitioned into training (80%) and test (20%) sets. To mitigate phylogenetic leakage, isolates were clustered using agglomerative hierarchical clustering on pairwise Hamming distances. A unified Hamming distance threshold of 0.05 was applied for clade-aware partitioning via AgglomerativeClustering and GroupShuffleSplit, following the theoretical framework for F1-optimal thresholding (Lipton *et al.* 2014). The decision threshold for inference was independently set to 0.05 based on the observed distribution shift in resistance prevalence between the internal and external cohorts. Specifically, the threshold was not optimized on training or internal test data; instead, it was determined empirically on the external validation cohort to account for covariate shift. A sensitivity analysis across six threshold values (0.05, 0.10, 0.20, 0.30, 0.40, 0.50) confirmed that 0.05 yielded the highest F1-score and MCC on the external cohort, while higher thresholds resulted in substantially reduced Recall (Table 3, available as supplementary data at *Bioinformatics Advances* online). Hyperparameter tuning was formulated as a multi-objective optimization problem jointly maximizing MCC and F1-score to minimize false negatives (Karl *et al.* 2023, Saleem *et al.* 2025, Habiba and Ludwig 2026). A Pareto-optimal configuration was identified using the Non-dominated Sorting Genetic Algorithm II (NSGA-II), Tree-structured Parzen Estimator (TPE), and Covariance Matrix Adaptation Evolution Strategy (CMA-ES) samplers. Model development and optimization were performed exclusively on the training portion; the external cohort (*n = *199) remained completely unseen during all stages. Performance was measured using accuracy, precision, recall, F1-score, MCC, and AUROC. Statistical significance was assessed via permutation testing (100 label shuffles). Non-parametric bootstrap resampling (1000 iterations) was applied to derive 95% confidence intervals (CI) for MCC, AUROC, and F1-score.

#### 2.3.1 Proposed hybrid framework: TabTransformer-CatBoost architecture

Hence, our proposed architecture is a multi-stage hybrid system that aims for capturing higher-order dependencies in genomic data via a hierarchical representation learning method. The framework consists of a Transformer-based feature encoder coupled with a gradient-boosted decision tree (GBDT) classifier. We set the embedding dimension of TabTransformer encoder to 32, four attention heads as well as three transformer layers (dropout = 0.2). The model was trained with Adam for 120 epochs (learning rate = 1 × 10^-^³, weight decay = 1 × 10^−4^, batch size = 128). All experiments were performed using a fixed random seed (42). Two hundred fifty boosting iterations of CatBoost were conducted to train the algorithm. Hyperparameters (depth, learning rate, L2 regularization) were fine-tuned using Optuna with 50 trials on the training set only.

#### 2.3.2 Feature representation and latent embedding

Traditional machine learning approaches treat genomic features as independent scalar values, failing to model the epistatic landscape. Each *K.pneumoniae* isolate is represented as a sparse binary vector x_i∈ {0,1}^*p*, where *p* = 195 denotes the number of genomic markers and each entry indicates the presence or absence of a specific AMR determinant.

To capture higher-order dependencies among genomic features, we implemented a contextual embedding layer inspired by the TabTransformer architecture. Each feature index *j* was associated with a learnable embedding vector


$$e_{j}\;\in \;R^{d}$$

representing feature identity. The binary value of each feature is projected into the same latent space and combined with its feature embedding:


$$z_{ij}=e_{j}+w_{v}x_{ij}$$

where $w_{v}$ learnable projection matrix mapping scalar feature values into the embedding space. The resulting token matrix


$$z_{i}\in \;R^{\mathrm{pxd}}$$

serves as input to a multi-head self-attention encoder. Self-attention updates each feature representation by incorporating contextual information from all other genomic markers:


$$\mathrm{Attention}(Q,K,V)=\mathrm{softmax}(\frac{QK^{T}}{\sqrt{d_{k}}})V$$

After passing through multiple transformer encoder layers, the contextualized feature representations are aggregated via mean pooling to obtain a single isolate-level embedding:


$$h_{i}=\frac{1}{p}\sum_{J=1}^{P}z_{ij}$$

This latent embedding summarizes the global genomic context of each isolate and serves as the input data for the downstream CatBoost classifier for resistance prediction. This embedding approach is consistent with transformer-based tabular representations (Luetto *et al.* 2025) and context-aware gene embedding frameworks (Minai *et al.* 2025).

#### 2.3.3 Downstream classification via CatBoost

CatBoost was applied to the transformer-derived latent embeddings rather than raw genomic markers. Unlike other GBDT implementations such as XGBoost or LightGBM, CatBoost does not require manual encoding methods that increase dimensionality and sparsity but instead implements a proprietary algorithm known as Ordered Boosting to process categorical data. As demonstrated by Prokhorenkova *et al.* (2018), this method serves as a useful deterrent against the “prediction shift” and the leakage of the target. Formulated from latent representation *z*, the final decision function *F*(*z*) is formed by an ensemble of *M* decision trees:


$$F(z)=\sum_{m=1}^{M}\gamma_{m}h_{m}(z)$$

This results in superior generalization performance on datasets where categorical interactions are dominant, such as bacterial resistomes, by ensuring unbiased gradient estimation during training.

#### 2.3.4 The composite hybrid mapping function

The proposed hybrid framework is mathematically defined as a composite mapping P=f ° Φ, where:


$$\Phi :X\;\to R^{L}$$

denotes the TabTransformer feature extractor that generates high-dimensional latent embeddings z from raw binary genomic data $X\;\in \;{\left\{0,1\right\}}^{195}$. This stage performs the structural interpretation of the resistome. Subsequently,


$$f:R^{L}\to [0,1]$$

represents the CatBoost classifier that performs the final phenotypic prediction. This hybrid synergy aims to overcome the limitations of individual models by separating representation learning from decision boundary optimization.

The mapping can be formally expressed as the minimization of the empirical risk:


$$L=\sum_{i=1}^{N}l(y_{i}f(\Phi \;(x_{i};\theta_{\Phi \;});\theta_{f}))$$

where $\theta_{\Phi \;}$ and $\theta_{f}\;$are the parameters for the Transformer and CatBoost components, respectively. The full mapping between all 32 latent embedding dimensions and original genomic features is provided in Table 1, available as supplementary data at *Bioinformatics Advances* online.

## 3 Results

### 3.1 Model performance on discovery cohort (internal benchmarking)

In this article, we used the high-fidelity discovery dataset (*n = *554 unique genomes) established through the preprocessing proposed in Section 2.1 and evaluated the predictive performance of a number of machine learning architectures utilizing a clade-aware hold-out validation strategy to avoid phylogenetic leakage.

#### 3.1.1 Base classifier benchmarking and selection

As Table 2 shows, initial benchmarking results indicate that resistance prediction from curated genomic markers is feasible using conventional models, with Logistic Regression achieving a competitive MCC of 0.7093. In the baseline state, CatBoost yielded the highest AUROC (0.9135) among the tree-based methods, demonstrating superior ranking performance. Consequently, due to its Ordered Boosting mechanism providing stable gradient estimation and effectively reducing prediction shift when working on high-dimensional tabular data, CatBoost served as the downstream classifier for our hybrid framework.

**Table 2:** Base classifier benchmarking.

Model	Accuracy	Precision	Recall	F1-Score	MCC	AUROC	Perm_MCC
LR	0.8951	0.9000	0.6585	0.7606	0.7093	0.9032	0.0070
Baseline CatBoost	0.8642	0.8800	0.5366	0.6667	0.6160	0.9135	0.0019
MLP	0.8333	0.8182	0.4390	0.5714	0.5152	0.8053	−0.0062
XGBoost	0.8086	0.7778	0.3415	0.4746	0.4267	0.8304	−0.0031
RF	0.7654	1.0000	0.0732	0.1364	0.2360	0.9219	0.0054

#### 3.1.2 Impact of hybrid architecture and representation learning

Additional analyses to capture non-linear dependencies and contextual genomic relationships focused on the tree-based and hybrid architectures using transformer-derived embeddings (Khalid *et al.* 2025). CatBoost was chosen as the downstream classifier because of its ordered boosting mechanism and stable estimation of gradient, which reduces the prediction shift (Prokhorenkova *et al.* 2018). In Table 3, all performance metrics for all those evaluated models are shown briefly. The self-attention encoder substantially enhanced predictive power, with MCC improving from 0.6160 (base model) to above 0.80 in hybrid models, a gain driven by representation learning rather than hyperparameter tuning, as extensively optimized standalone CatBoost models failed to match hybrid performance. Of the configurations tested, the Chained Hybrid configuration produced (AUROC = 0.8670, F1 = 0.8537) the highest diagnostic reliability. A second iteration of the model was fitted, with the NSGA-II alongside TPE and CMA-ES, for comparison. Curiously, the performance of all search strategies converged toward almost identical optimal configurations. This convergence further demonstrates the robustness of the learned genomic embeddings.

To further justify the choice of CatBoost as the downstream classifier, ablation experiments were conducted using the same transformer-derived embeddings with alternative classifiers: Logistic Regression (Embedding + LR), a multilayer perceptron (Embedding + MLP), and a standalone Transformer-Only model with a built-in classification head. Among these, Embedding + MLP achieved MCC = 0.786 and Embedding + LR achieved MCC = 0.768, both falling below the Chained Hybrid (MCC = 0.804). The Transformer-Only baseline yielded the weakest performance (MCC = 0.728), indicating that the self-attention encoder alone is insufficient and that the gradient boosting classifier contributes substantially to the final predictive gain. Finally, permutation MCC values remained near zero across all models (Table 3), statistically confirming that predictive performance was not driven by random label structure (mean Perm MCC ≈ 0.00, *P* < .01).

**Table 3:** Impact of hybrid architecture and representation learning test result.

Model	Accuracy	Precision	Recall	F1-score	MCC	AUROC	Perm MCC
Chained hybrid	0.9259	0.8537	0.8537	0.8537	0.8041	0.8670	−0.0020
TabTransformer-CatBoost	0.9259	0.8718	0.8293	0.8500	0.8013	0.8702	0.0119
Embedding + MLP	0.9198	0.8500	0.8293	0.8395	0.7861	0.8742	−0.0044
Embedding + LR	0.9136	0.8462	0.8049	0.8250	0.7681	0.8718	0.0139
Transformer-only	0.9012	0.8788	0.7073	0.7838	0.7279	0.8879	0.0056
Optimized CatBoost TPE	0.9012	0.8788	0.7073	0.7838	0.7279	0.9022	0.0047
Optimized CatBoost CMA-ES	0.8951	0.8333	0.7317	0.7792	0.7134	0.8982	−0.0056
Optimized CatBoost NSGA-II	0.8951	0.8529	0.7073	0.7733	0.7111	0.9014	0.0048
Baseline CatBoost	0.8642	0.8800	0.5366	0.6667	0.6160	0.9135	0.0019

### 3.2 External validation and generalizability

The Chained Hybrid framework's utility as a clinical treatment tool was validated externally on 199 unique genomic signatures (derived from 305 initial isolates) across seven distinct BioProjects. As shown in Table 4, under the theoretical background for optimal thresholding based on non-calibrated or shifted distributions, a fixed decision threshold of 0.05 was used to avoid loss under covariate shift.

**Table 4:** External validation performance of the chained hybrid model across seven independent BioProjects.

Metric	Value
Accuracy	0.7279
Precision	0.8258
Recall (sensitivity)	0.6957
F1-score	0.7552
MCC	0.4638
AUROC	0.8105
Perm MCC	−0.001

The model, at this calibrated operating point, had consistent external predictive performance with AUROC of 0.8105, F1-score of 0.7552, and precision of 0.8258. Although absolute values were only slightly lower than in the internal evaluation, the retention of AUROC and F1 across heterogeneous cohorts suggests that the learned embedding space preserves resistance-associated genomic signals against significant differences between datasets. These observed results can confirm the scalability of the implemented hybrid model in the real world with validation. Nonetheless, the decrease in MCC (0.8041 in internal to 0.4638 in external) corresponds to the anticipated effect of cross-cohort heterogeneity and showcases the struggle for accurate prediction of resistance in different genomic backgrounds.

### 3.3 Diagnostic performance and error analysis

**Figure 2:**
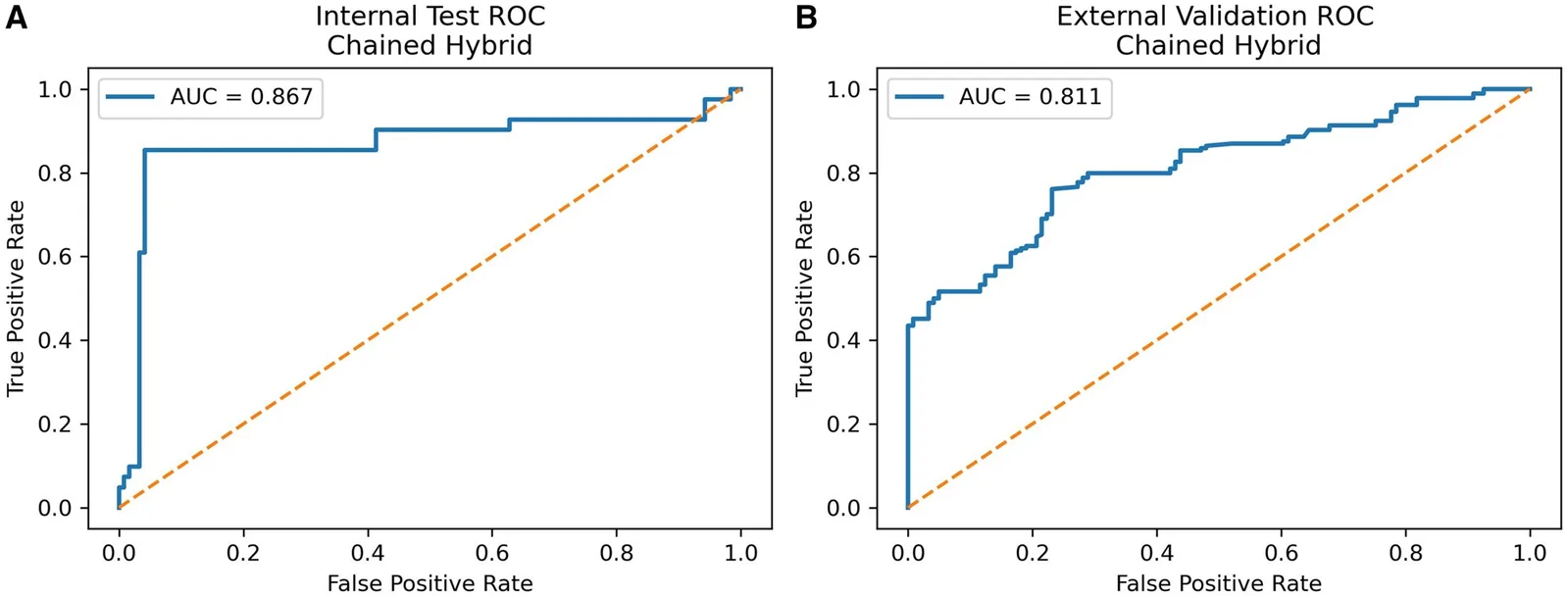
Receiver operating characteristic curves for the Chained Hybrid model using transformer-derived embeddings. (A) Internal test evaluation. (B) External validation across independent BioProjects.

**Figure 3:**
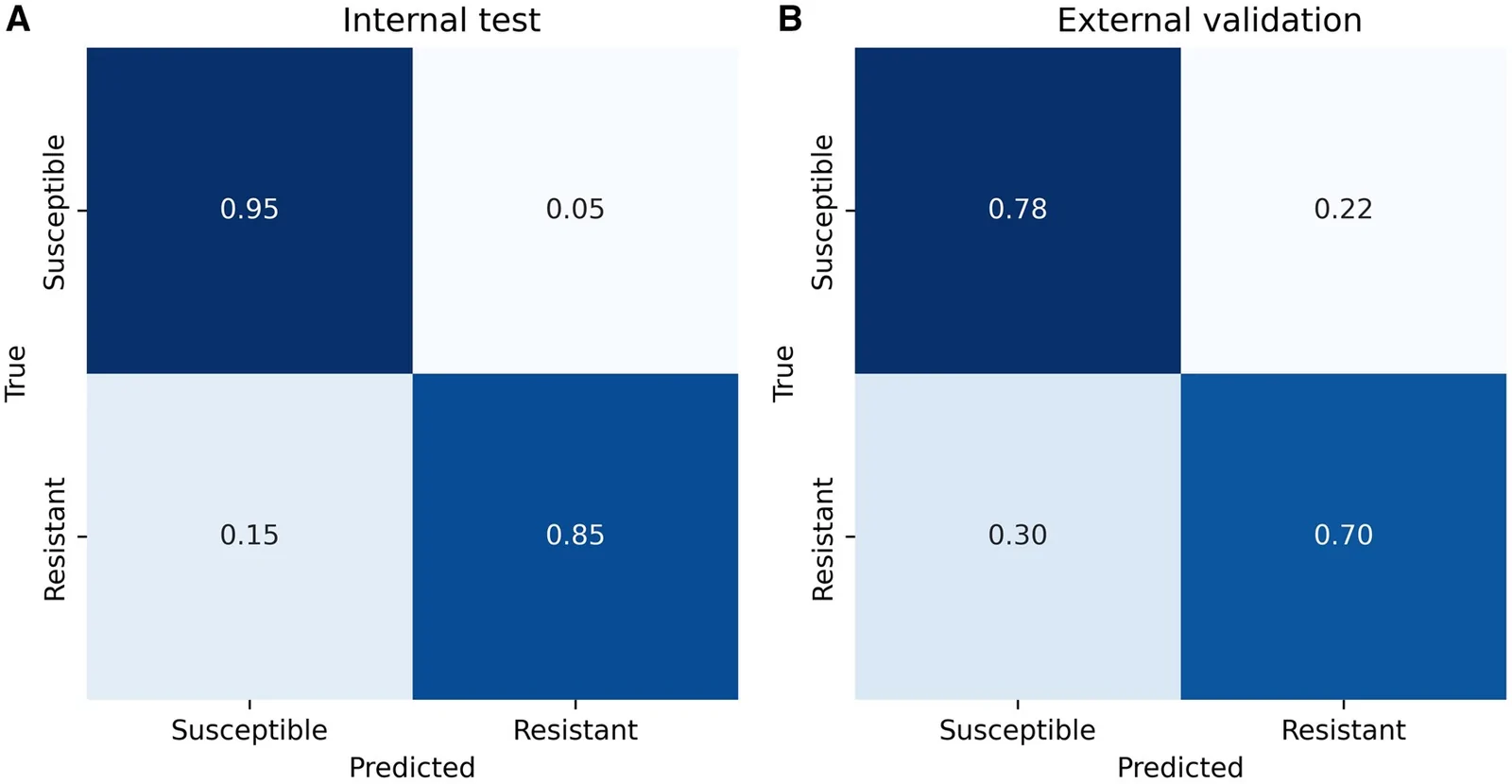
Confusion matrices for the proposed hybrid model. (A) Internal clade-aware test set. (B) External multi-cohort validation.

As shown in Figs 2 and 3, the model demonstrates strong discriminative performance under internal evaluation and retains predictive capacity under cross-project validation, although reduced performance indicates distributional heterogeneity across cohorts.

Values are row-normalized. The model preserves high detection of resistant isolates while maintaining precision across heterogeneous datasets. The confusion matrix analysis further demonstrates that the hybrid framework maintains high sensitivity for resistant isolates, thereby reducing clinically consequential false-negative predictions under both internal and external evaluation settings.

### 3.4 Latent space visualization and feature interaction

Transformer attention analysis demonstrated a well-defined interaction landscape between resistance-associated genomic features. To assess the encoder’s recognition of coordinated signals, we extracted averaged multi-head self-attention weights across transformer layers and ranked the strongest pairwise interactions among genomic markers. This results in a unique interaction profile of resistance associated loci with co-attention patterns, suggesting that the model learning is co-observed joint genomic signatures rather than relying on gene presence alone. These attention generated interactions are further indicative of the way the transformer jointly weights genomic features during representation learning. A few β-lactamase and efflux related markers showed consistently elevated co-attention scores, which suggests that resistance prediction depends on synchronized feature clusters that are encoded in the latent representation. Although attention weights do not imply causality by biology, stability of the top feature pairs from one to another indicates that the model accounts structured co-occurrence patterns associated with meropenem resistance.

**Figure 4:**
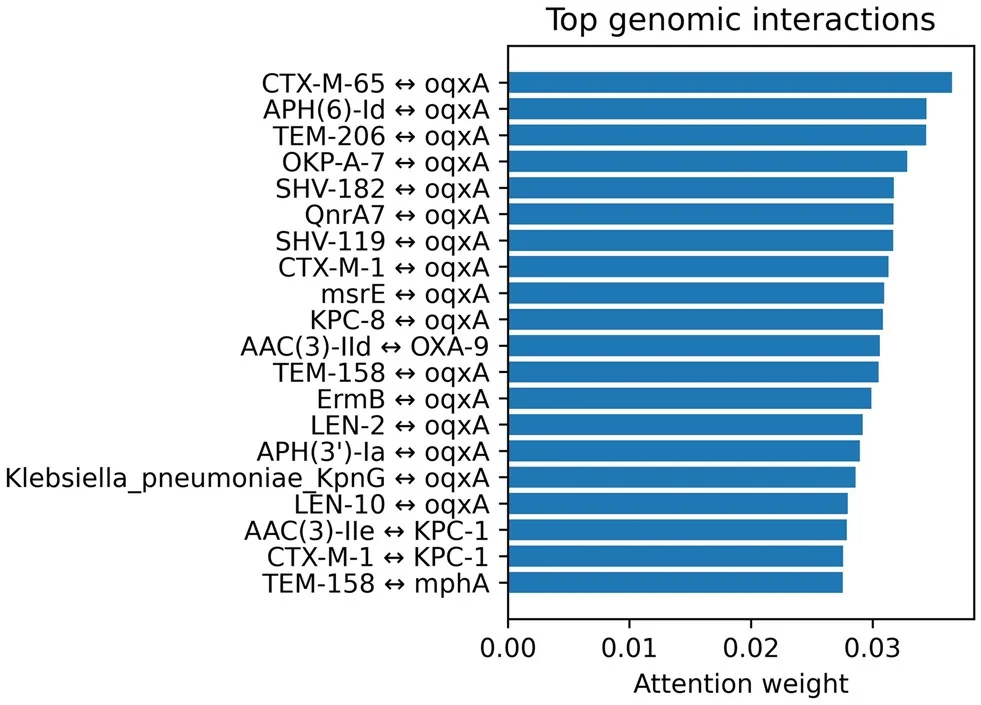
Top genomic interactions captured by transformer self-attention. Attention weights reflect contextual importance rather than direct causal biological interaction.

Importantly, oqxA, a plasmid-borne efflux pump gene, emerged as the dominant interaction hub, co-attending with 16 of the top 20 gene pairs, including CTX-M-65, SHV-182, QnrA7, and KPC-8 (Fig. 4). Such results are epidemiologically consistent with previously published surveys reporting oqxAB prevalence of 100% among KPC-producing sequence type 258 (ST258) *K.pneumoniae* isolates, the dominant lineage in the Houston collection (Perez *et al.* 2013, Wyres and Holt 2018). The oqxAB efflux pump, chromosomally encoded but also transferable via large plasmids, confers co-resistance across multiple antibiotic classes including fluoroquinolones and contributes to the broader MDR phenotype observed alongside carbapenemase genes (Li *et al.* 2019). Its emergence as the central attention hub in our model suggests that the transformer captures not only carbapenem-specific resistance signals but also the broader genomic context of multidrug resistance dissemination in clinical *K.pneumoniae*. The presence of two other high-weight pairs, AAC(3)-IIe with KPC-1 and CTX-M-1 with KPC-1, further indicates that the model jointly encodes aminoglycoside and ESBL co-resistance signals alongside carbapenem resistance.

### 3.5 Model interpretability and feature attribution

**Figure 5:**
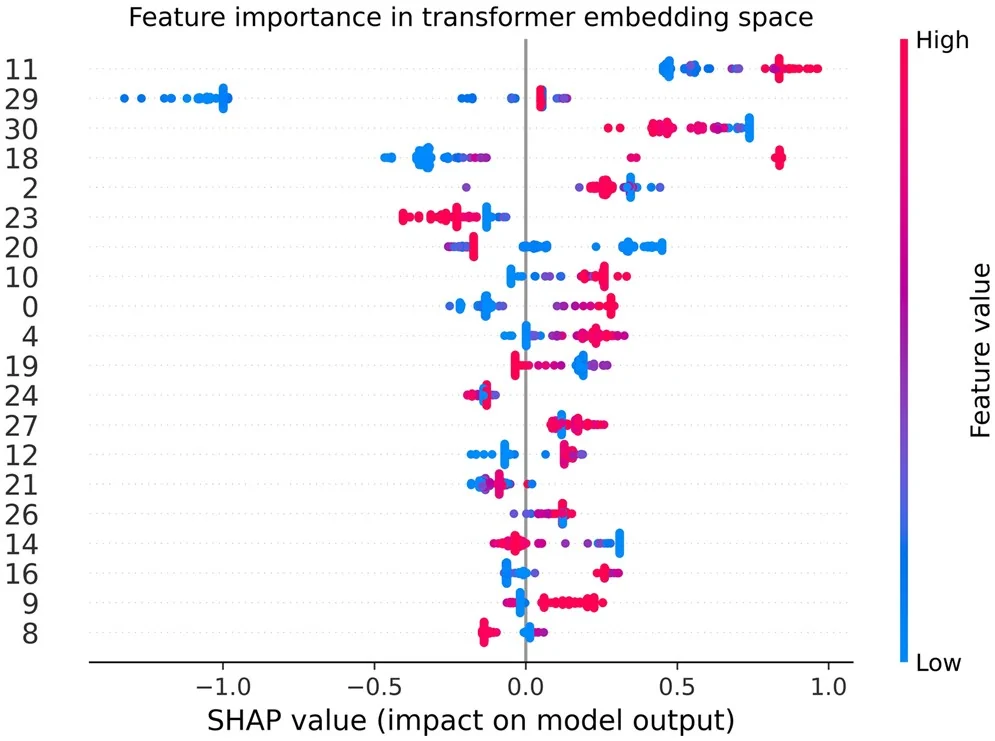
Feature importance in transformer embedding space. SHAP summary plot of the 32 latent embedding dimensions for the Chained Hybrid model. Each point represents a training sample; color indicates feature value (red: high, blue: low).

To examine the biological relevance and structural properties of the acquired representations, we conducted interpretability analysis based on embedding-space SHapley Additive exPlanations (SHAP) attribution and latent space visualization. Rather than directly attributing importance to genes, SHAP values were calculated on the transformer-derived embedding vectors used as input for the CatBoost classifier (Lundberg and Lee 2017). A number of the embedding components had consistently positive SHAP contributions for resistant isolates, indicating that prediction is driven by structured patterns in the learned representation rather than isolated gene presence–absence signals (as is evident in Fig. 5). The distributed importance across multiple embedding dimensions suggests that meropenem resistance is modeled as a coordinated genomic signature rather than a single dominant determinant. For resistant isolates, dimension 11 provided the highest positive SHAP contribution among individual dimensions, whereas dimension 29 presented the strongest negative deviation, indicating that it encodes a susceptibility-associated genomic context. The majority of remaining dimensions clustered near zero, confirming a distributed rather than sparse representation.

To address the interpretability gap from the latent space to microbial genetics, we conducted a global correlation analysis across all 32 embedding dimensions. Three main determinants dominate the interpretability analysis, representing a hierarchical genomic fingerprint. BlaKPC-1 is still the primary driver (|*r*| = 0.91) and a top feature in all 32 latent dimensions. Then comes blaOXA-9 (|*r*| = 0.66) and blaTEM-122 (|*r*| = 0.41), representing significant concurrent markers. This triad consists of the most basic elements of the model’s “semantic map,” as the self-attention mechanism exploits their combined presence to differentiate carbapenem-resistant isolates from susceptible isolates with high accuracy (Table 5).

**Table 5:** Hierarchical genomic signature mapping.

Gene name	Avg. correlation (|*r*|)	Biological role	Clinical significance
blaKPC-1	0.91	Primary resistance driver	Central determinant of carbapenem resistance in *K.pneumoniae*.
blaOXA-9	0.66	Primary co-occurring marker	Key synergist often found on the same mobile genetic elements as blaKPC.
blaTEM-122	0.41	Secondary resistance modulator	Auxiliary beta-lactamase contributing to the broader epistatic resistance landscape.

**Figure 6:**
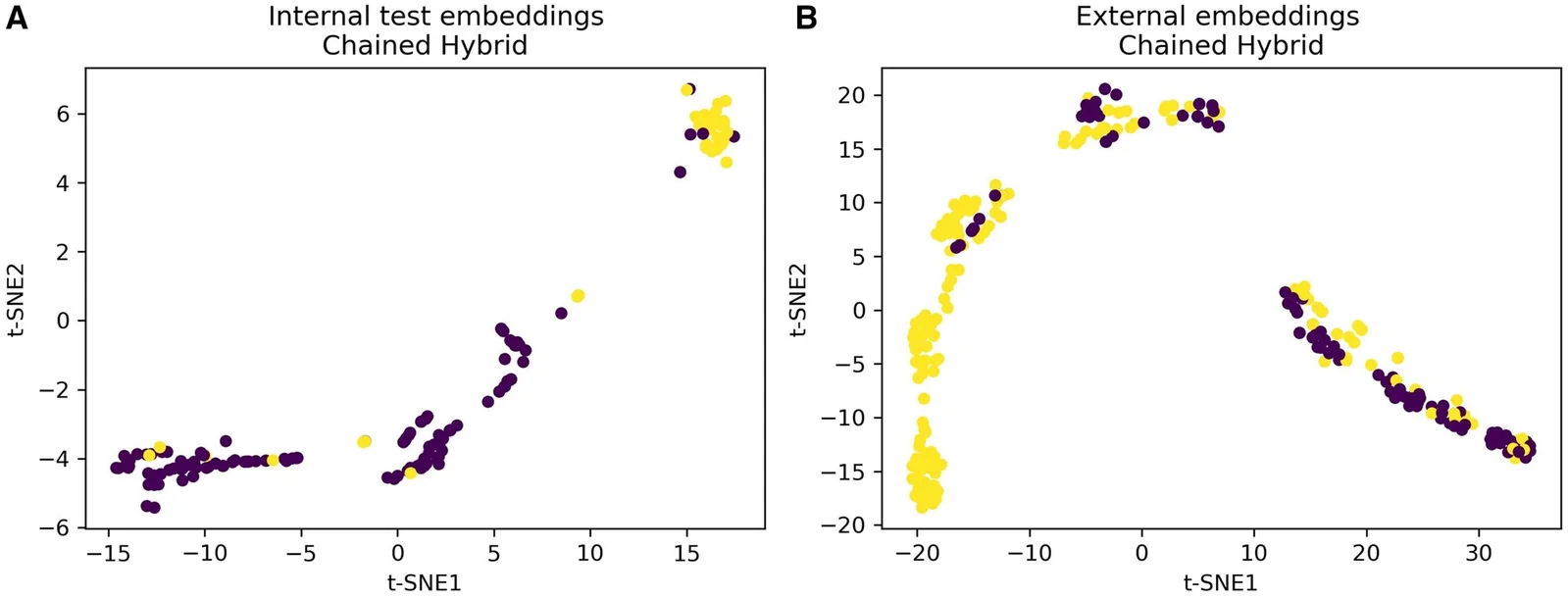
t-SNE visualization of transformer-derived genomic embeddings. (A) Internal clade-aware test set. (B) External multinational validation cohort. Resistant and susceptible isolates are indicated by distinct marker colours.

For deeper exploration of the geometry of the learned representation, we visualized transformer-derived embeddings using t-SNE dimensionality reduction (Fig. 6). The internal clade-aware test set showed distinct differentiation between resistant and susceptible isolates in the latent space, suggesting that the embedding layer encodes the phenotype-associated genomic structure, beyond the original binary gene matrix. Such partial overlap between classes in the external cohort was observed, especially in the lower-left embedding region, but two spatially distinct resistant subclusters were still visualizable in the external space, indicating that the learned representation maintained phenotype-associated structure across heterogeneous genomic sources even in the face of cross-cohort distributional change (Fig. 6). This aligns with the hypothesis that self-attention mechanisms account for transferable genomic underpinnings of resistance but still accurately reflect true cross-cohort heterogeneity.

**Figure 7:**
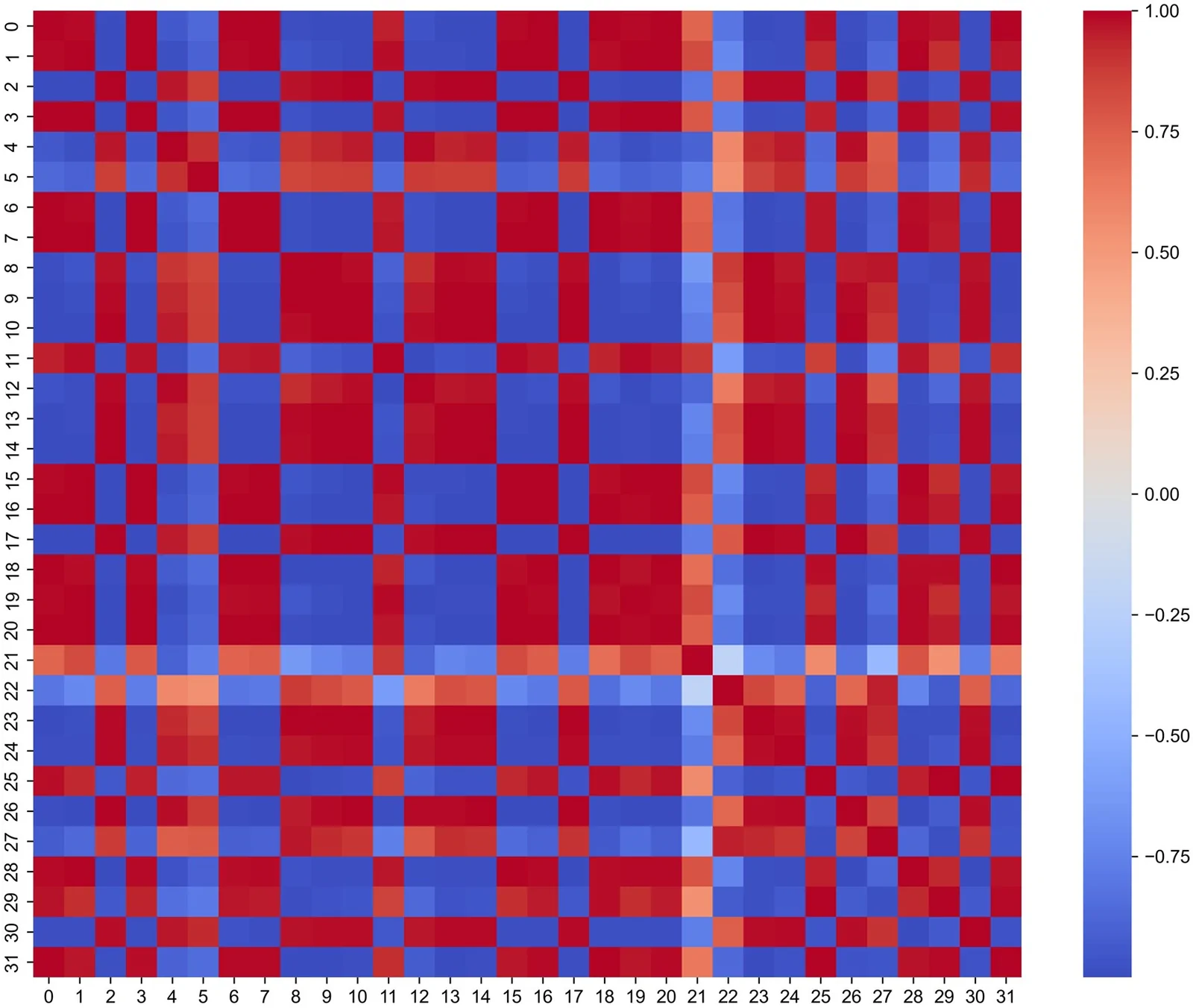
Pairwise correlation between latent embedding dimensions.

All together, these analyses demonstrate that the predictive power of the hybrid framework emerges mainly from the representation learning phase. The transformer encoder constructs a biologically structured embedding space to be later exploited by the gradient-boosted classifier for robust resistance prediction. To investigate the structural independence of the learned embedding dimensions, we carried out pairwise Pearson correlations for all 32 latent components. The correlation heatmap obtained in Fig. 7 showcases an obvious checkerboard form, characterized by systematic anti-correlations (*r* ≈ −0.75) on alternate embedding dimensions. This network structure shows that the transformer learned complementary encoding pairs—if one dimension encodes a resistance-related signal, its partner encodes the contrasting susceptibility context. A block of strong positive correlations from dimensions 20 to 24 indicates a shared resistance-encoding subspace, where the other dimensions maintain partial orthogonality as in distributed genomic representation.

### 3.6 Ablation study: assessment of information leakage and data redundancy

To assess the effect of information leakage and a lineage-driven bias on information quality, we performed an ablation analysis. Using a standard random-split configuration, we compared our framework to a baseline model trained on the un-deduplicated, raw dataset. In those conditions, a standalone CatBoost model obtains an inflated internal accuracy of 91.73% and an AUROC of 0.9262. This good performance is usually due to identical genomes occurring in both of the partitions. It results in model memorization rather than true biological generalization (James *et al.* 2025, Malajczuk *et al.* 2026). These distinctions were crucial to external validation. The raw model had severe generalization collapse when it was applied to the independent cohort. The recall fell to 22.28% and Matthews Correlation Coefficient to 0.3196. This staggering discrepancy suggests that when models are trained on redundant signatures they generally represent lineage-specific trends. In contrast, our proposed hybrid framework exhibits much higher stability across heterogeneous backgrounds. This stability indicates that the predicted meropenem resistance is indeed based on genuine epistatic signals.

## 4 Discussion

A key point of this study is that transformer-formulated embeddings can notably enhance resistance prediction under leakage-aware genomic partitioning. By mapping out the genomic similarity cutoff to the probability decision boundary, the model normalized sensitivity to resistance-related signals while reducing phylogenetic leakage. This approach is in line with the proposed notion of the optimal threshold of F1 (Lipton *et al.* 2014). Also, transformer-inspired embeddings based on the self-attention architecture defined by Vaswani *et al.* (2017) yielded significantly greater predictive performance than classical models. The baseline CatBoost model achieved a higher AUROC = 0.9135, whilst its lower Recall and MCC demonstrate that its advantage for global ranking did not lead to balanced classification. A DeLong test showed that the difference in AUROC was statistically significant (*P* = .022). Meanwhile, the embeddings improved threshold-dependence measurements of the model, such as MCC of 0.8041 and F1-score of 0.8537. The observed increase reflects a better decision boundary calibration and minority class sensitivity. Bootstrap resampling also demonstrated the statistical stability of the model. Internal tests gave an MCC of 0.8032 (95% CI: 0.6875–0.8969) and AUROC of 0.8673 (95% CI: 0.7826–0.9438). External validation revealed a muted decrease in effectiveness with an AUROC of 0.8115 (95% CI: 0.7614–0.8565) and MCC of 0.4638 (95% CI: 0.3605–0.5645). Yet, the CI were well in excess of zero. The observed reduction in MCC from 0.8041 (internal) to 0.4638 (external) is attributable to cohort heterogeneity across the external validation dataset. Several sub-cohorts consisted entirely of susceptible isolates or represented single endemic lineages, precluding balanced metric computation and contributing disproportionately to aggregate performance reduction. The geographic diversity of the external cohort, spanning isolates from the USA, Pakistan, Italy, and Greece, introduces lineage-specific resistance gene distributions not fully represented in the internal training data. These findings suggest that the MCC reduction reflects feature distribution shift across geographically distinct collections rather than a fundamental limitation of the learned genomic representation. The embedding dimension (32) was selected conservatively for the feature space (195) due to the bias of overfitting on the deduplicated cohort. The TabTransformerExtractor comprises approximately 32 002 trainable parameters, a deliberately compact architecture relative to conventional large-scale transformers, enforcing a compression ratio of approximately 0.16 and producing a regularized latent representation. Overfitting risk was further mitigated through dropout (*P* = .20) and L2 weight decay (*λ* = 1 × 10^−4^). It is also important to note that the transformer serves exclusively as a feature extractor; the final classification is performed by CatBoost, which provides additional robustness through its ordered boosting mechanism. Embedding stability was corroborated by the convergence of three independent hyperparameter optimization strategies (TPE, CMA-ES, NSGA-II) toward near-identical optimal configurations, indicating that the learned embedding space is consistent and not sensitive to stochastic initialization. Non-parametric bootstrap resampling using standard statistical concepts were followed as appropriate (Efron and Tibshirani 1994).

Recent research (Condorelli *et al.* 2024) show that traditional Gradient Boosting is accurate, our hybrid design is consistent with the nature of structured epistatic content. Correlation mapping revealed a hierarchical genomic signature, which is dominated by three markers (blaKPC-1, blaOXA-9, and blaTEM-122). This signature fits epidemiologically with the Houston collection (PRJNA376414) where resistance to ST258 was driven by the lineage. As shown by Kavvas *et al.* (2018), significant genetic features can be successfully detected in a pan-genome using computational frameworks, supporting that DNA-based models capture clinically relevant resistance signals without transcriptomic layers. These findings align with attention-based genomic embedding frameworks for phenotype inference (Huang *et al.* 2020, Choi and Lee 2023). Using NSGA-II for multi-objective optimization, we found Pareto optimal configurations combining precision with recall (Verma *et al.* 2021, Ma *et al.* 2023). The t-SNE and SHAP analyses reflect current XAI paradigms (Kim *et al.* 2022, Sufi 2025). Permutation testing and ablation analysis jointly affirm that both leakage-aware deduplication and clade-aware partitioning are critical to reliable prediction of AMR. Notwithstanding these strengths, several limitations of the present study warrant acknowledgement. The discovery cohort comprised 554 unique genomes following leakage-aware deduplication, which may limit generalizability to underrepresented sequence types. The framework was developed and validated exclusively for meropenem resistance in *K.pneumoniae*, and its applicability to other antimicrobial classes or ESKAPE pathogens remains to be established. Sub-cohorts with limited class representation in the external dataset precluded reliable computation of discrimination metrics, highlighting the dependency of genomic prediction models on balanced phenotypic surveillance data. Finally, clinical deployment would require prospective validation across diverse sequencing platforms and assembly pipelines to confirm robustness beyond the retrospective WGS datasets employed here.

## 5 Conclusion

In this article, we presented a novel hybrid computational framework, TabTransformer-CatBoost, designed to predict meropenem resistance in *K.pneumoniae* with high diagnostic precision. Multi-objective optimization ensured clinically reliable performance by prioritizing false-negative minimization. Experimental results validated on an independent cohort demonstrate that gene-based models can achieve strong predictive performance (AUROC = 0.8670; F1 = 0.755), providing a rapid and scalable alternative to time-consuming phenotypic assays. Latent-space analysis and correlation-based mapping further indicate that the model captures key resistance determinants such as blaKPC while accounting for their broader genomic context. Future work will evaluate the framework across additional ESKAPE pathogens, antimicrobial classes, and prospective clinical sequencing cohorts. Expanding training cohort size through federated learning or transfer learning approaches may further improve generalizability across underrepresented sequence types and geographic regions. By integrating contextual genomic embeddings with leakage-aware evaluation, the proposed framework provides a robust foundation for future data-driven AMR prediction systems in clinical genomics.

## Supplementary Material

vbag193_Supplementary_Data

## Data Availability

The genomic data used in this study are publicly available through the Bacterial and Viral Bioinformatics Resource Center (BV-BRC). The primary discovery cohort is deposited under NCBI BioProject PRJNA376414. External validation cohorts are available under BioProjects PRJEB6574, PRJNA288601, PRJNA278886, PRJNA307517, PRJNA296771, PRJEB22890, and PRJNA312972. The preprocessed training dataset (kp_meropenem_training.csv) and external validation dataset (kp_meropenem_external_validation.csv) are publicly available on Zenodo (https://doi.org/10.5281/zenodo.20391155). The source code for the TabTransformer-CatBoost framework, including fully documented pipeline scripts, dependency files, and a contribution guide, is available at https://github.com/SibelKervanci/kp-meropenem-tabtransformer.
